# Competition limits adaptation and productivity in a photosynthetic alga at elevated CO_2_

**DOI:** 10.1098/rspb.2010.1173

**Published:** 2010-08-04

**Authors:** Sinéad Collins

**Affiliations:** Institute of Evolutionary Biology, University of Edinburgh, Kings Buildings, Ashworth Laboratories, West Mains Road, Edinburgh EH9 3JT, UK

**Keywords:** adaptation, competition, carbon dioxide, *Chlamydomonas*, Price equation, community productivity

## Abstract

When competitive exclusion between lineages and genetic adaptation within lineages occur on the same timescale, the two processes have the potential to interact. I use experimental microbial evolution where strains of a photosynthetic microbe that differ in their physiological response to CO_2_ enrichment are grown either alone or in communities for hundreds of generations under CO_2_ enrichment. After about 300 generations of growth, strains that experienced competition while adapting to environmental change are both less productive and less fit than corresponding strains that adapted to that same environmental change in the absence of competitors. In addition, I find that excluding competitors not only limits that strain's adaptive response to abiotic change, but also decreases community productivity; I quantify this effect using the Price equation. Finally, these data allow me to empirically test the common hypothesis that phytoplankton that are most able to take advantage of carbon enrichment in single-strain populations over the short term will increase in frequency within multi-strain communities over longer timescales.

## 1. Introduction

Microbes have short generation times and large population sizes, so that on the timescale of decades, microbial communities will respond to environmental change at multiple levels simultaneously. Concretely, the individual components of microbial communities will respond to environmental change physiologically through changes in phenotype without any underlying change in genotype, as well as evolutionarily through genetic change within lineages [1]. At the same time, the composition of microbial communities will change as competitive interactions shift. All three levels of response (physiology, evolution and competition) can occur together, so that explaining changes in population characters, such as productivity, requires that we untangle the effect of adaptation within strains to abiotic environmental change from the effect of competition between strains. Here, I use experimental evolution in simple communities of a photosynthetic microbe to investigate the general interaction between competitive and non-competitive evolutionary responses to CO_2_ enrichment over hundreds of generations in a case where the two types of responses occur simultaneously, and to show the effect of competition on the fitness and productivity of the evolved communities.

The main goal of this selection experiment is to examine the general interaction between the ability to adapt to abiotic change in the absence of competitors and the ability to exclude competitors when they are present during that same environmental change. I use a particular environmental change (elevated CO_2_) that allows me to test two basic assumptions that are commonly used to make qualitative predictions about how phytoplankton communities may respond to global change. The first assumption is that types with the most pronounced physiological response to CO_2_ enrichment measured in pure culture should increase in frequency in communities [2]. This supposes that the ability to respond to CO_2_ enrichment by increasing growth rate or biomass rapidly (without genetic change) will result in a competitive advantage over longer timescales, and that measurements made in pure cultures can be used as predictors of competitive success. The second assumption is that physiological responses to CO_2_ enrichment measured in single-lineage cultures inform us about the productivity of the end community made up of those lineages [3–5]. To this end, I started selection experiments with three different genotypes of *Chlamydomonas reinhardtii* that differ in their carbon uptake or carbon fixation.

## 2. Material and methods

### (a) Selection experiment

Three strains with different carbon uptake and carbon fixation capabilities were used. The first two strains are a wild-type strain (2137) and a Rubisco mutant (D473E, simply called 473 here), provided by R. Spreitzer and described by Satogonpan & Spreitzer [6]. The Rubisco mutant can take carbon up normally, but fixes it slowly. The wild-type 2137 takes up and fixes carbon normally. The third strain is a mutant strain (cc2699mt+, simply called 2699 here) with a compromised copy of Cah3, a gene that encodes a carbonic anhydrase that is part of the *Chlamydomonas* carbon concentration mechanism (CCM) [7]. 2699 was obtained from the *Chlamydomonas* centre culture collection. This mutant has an altered CCM, but normal carbon fixation. I measured the initial fitnesses (± s.e.m) of the three strains in air and at high CO_2_ as follows, in number of cell doublings per 7 days: 473: 13.41 ± 0.26, 2137: 13.69 ± 0.32, 2699: 17.38 ± 0.05. At high CO_2_: 473: 15.74 ± 0.12, 2137: 15.88 ± 0.14, 2699: 18.79 ± 0.02.

Five different communities were used. Three communities consisted of each strain grown alone and are called single-strain communities. Two communities were found with equal numbers of either the two mutants (473 and 2699) or all three strains. These are called multi-strain communities. The five communities are: (i) 2137 alone, (ii) 473 alone, (iii) 2699 alone, (iv) 473 and 2699 (community A), and (v) all three strains (community B). All communities were composed of mating type plus individuals only, and all reproduction was therefore asexual.

Each strain used was founded from a single clone so that all evolution within strains must use de novo mutations. Three independent replicate populations of each community were propagated for 22 transfers with 7 days between transfers, for a total of approximately 320 generations, under each of the following conditions: in air (control treatment), at elevated CO_2_ (high treatment) or in gradually rising CO_2_ (rising treatment). The high treatment was 1 per cent CO_2_. The rising treatment consisted of 20 equal step increases in CO_2_ levels, at the time of transfer, from transfers 2–21 inclusive. In the rising treatment, the initial CO_2_ level at transfer 1 was air, and the final level was 1 per cent, so that the magnitude of difference between the initial and final environments is the same in both the high and rising enrichment regimes; only the rate of enrichment differs. The goal in this experiment was to investigate the interplay of competitive exclusion and adaptation when they occur at the same time; only a single strain persists through to the end of the experiment under the conditions used.

*Culture and transfer details*: All cultures were grown in Suoka high salt media [8] in sterile 96-well microplates with 100 µl of medium per well. Absorbance at 650 nm for each plate was measured at the end of each 7 day growth period (just before transfer). A fixed proportion (one-tenth) of the population was transferred. For transfer, the contents of all wells from a single plate were pooled, diluted with sterile high salt media (HSM), New England BioLabs and then pipetted into a new sterile 96-well plate. The goal of this experiment was to examine how competition between strains interacts with adaptation to an abiotic change within strains when the two processes occur on the same timescale. Traditional, well-mixed microbial cultures model an ecological scenario where competition is very strong because no refugia are present in the environment, such that small differences in growth rates result in deterministic and rapid competitive exclusion. To model cases where competitive exclusion is slow enough to occur at the same rate as adaptive evolution within strains, I chose an ecological scenario where the plate provided a physically structured environment that slowed competitive exclusion, allowing adaptation and competition to occur on the same timescale. Competitive exclusion occurred between transfers 4 and 20 (between approx. 30 and 300 generations) in all but one of the replicate multi-strain communities. At every transfer, an aliquot of each population was stored on HSM agar plates. Aliquots for DNA extraction were taken every other transfer.

### (b) Community composition

Community composition (monomorphic versus polymorphic) of the multi-strain communities was monitored by PCR followed by restriction digestion. Genomic DNA was extracted using a modified HotSHOT method [9], where cells were vortexed with glass beads before and after hot NaOH extraction. Genomic DNA was amplified using the following primers: Cah3 gene fragment: left primer: 5′-CATGGGTCAACATGGGTTCT-3′, right primer: 5′-GCAACACCCACCATAGTTCC-3′. The Cah3 amplicon was digested with AlwN1 (New England BioLabs), which only cuts amplicons from 2699. Rubisco large subunit fragment: left primer: 5′-GCTGCATGTGAAGTTTGGAA-3′, right primer: 5′-GCACAGGCAAATTTAAACAAAA-3′. The Rubisco amplicon was digested with Mfe1 (NEB), which only cuts amplicons from 473. Ancestors and single-strain communities, both alone and mixed in known ratios, were used as controls for PCR and restriction digestion-based strain markers. These markers allow single-strain communities to be reliably distinguished from multi-strain communities, but cannot distinguish a three-member community from a two-member community using standard PCR.

### (c) Fitness and growth assays

This experiment tests the assumption that information measured in the absence of competitors, in cultures evolved as single strains in a new or changing environment, may be used to predict how those same strains will fare in that same environment when they are part of a community (see for example [2,5,10–13]). Because of this, the contributions to fitness from competition between strains and adaptation within them in response to abiotic change alone must be treated separately in this study. Since a measure of how fitness has changed in response to abiotic environmental change that is not based on competition is needed, the usual measure of fitness in microbial selection experiments (competitive fitness) is not appropriate here. Growth rate, one common measure of pure-culture fitness, is also not a good fitness proxy here, as populations have evolved diverse life-history strategies, so that differences in the number of offspring produced can be attributed to differences in one or more of lag time, growth rate and carrying capacity. To take this into account, I have used an integrated metric for fitness in the absence of competitors that reflects the ability of an asexual strain to produce offspring under the conditions of this particular experiment. Here, single-strain fitness is measured as the total number of cell divisions that had to occur to account for new cells produced in the time allotted between transfers (7 days). This is analogous to the number of offspring (or grand-offspring) produced by the strain. There are several non-exclusive ways to increase this metric for fitness by changing one or more life-history traits (lag time, growth rate, maximum cell density); all of these will affect the number of cells produced between transfers, so that the metric used here is an integrated measure of fitness in batch culture that accounts for the transfer regime where a fixed proportion of the population rather than a fixed number of cells was transferred between microcosms. Fitness (*n*) was calculated as the total number of cell divisions needed to account for the total amount of new cells that were produced over 7 days, where (final population size – initial population size) = 2^*n*^. Since the starting population is large, note that the number of cell divisions may appear high since many parents may contribute to the population, thus increasing the total number of cell divisions occurring in the culture. This metric allows a general measure of fitness that can be increased by changing one or more components of life histories, and does not penalize the strategy of simply being very productive. Because a fixed proportion rather than a fixed number of individuals from the population was transferred between microcosms, producing a large number of offspring by having a large number of parents is a valid strategy for an asexual single-strain population in batch culture. Imagine two populations of the same strain, one started with 10 times more cells than the other. Whatever the lag time, if both populations reach the same carrying capacity, then they must differ in the number of doublings they have undergone (log_2_(10) versus log_2_(100)). Thus, despite them both having the same carrying capacity, the calculated fitnesses would differ if they were only measured over a single growth cycle. However, this difference would be eliminated after this single growth cycle if they reach the same carrying capacity again, since both populations would now both contribute equal numbers to the next growth cycle (since they reached the same carrying capacity, and the same proportion of each population is transferred). Here, all fitness measurements were made after populations had already acclimated for a full growth cycle; differences in initial cell numbers during the growth cycle used to calculate fitness do contribute to fitness differences and are part of the growth strategy of the population. Note that growth rate, a conventional fitness proxy, was also measured and considered as a component of life history.

Fitness was measured in 96-well plates containing 100 µl of Suoka HSM in each well. Populations were first acclimated to the assay environment for 7 days, then diluted to a known cell density and used to inoculate fresh 96-well plates. Starting densities of cultures were similar to transfer densities used during the selection experiment. Since different cultures evolved different life-history strategies, they were at different points on the growth curve at the end of the 7 day acclimation period. This difference in life-history strategies is an integral part of differences in adaptation in a batch culture experiment, and so cultures were not forced to begin the fitness assay during a particular phase of growth, but rather allowed to use whatever strategy they had evolved, since there are many non-exclusive ways to increase fitness in batch culture. See table 1 for strain-specific information. Absorbance at 650 nm was measured every 24 h. Population growth curves were fit using JMP (SAS) with a three phase function [14], as previously published [15]. Independent triplicate cultures were grown for each population in each environment. Standard curves were plotted for each community at the beginning and end of the experiment; cell number relates to absorbance over the values used. When the physiological response is measured, it is the fitness of a community at high CO_2_ relative to the fitness of that same community in air.

**Table 1.** Outcome of competition and life-history traits of evolved communities. Summary of the outcome of competition and of life-history traits at high CO_2_ in evolved communities. Life-history traits are measured over 7 days. Populations were considered to have reached carrying capacity if they showed no increase in absorbance for 24 h. In all cases, communities were first acclimated for 7 days in high CO_2_ before the assays were carried out for the data shown here. All growth rates are relative to the wild-type (2137), when evolved in a single-strain community in the control regime and grown at ambient CO_2_. Values are the average ±s.d. of three independent communities. Between 8 and 12 independent replicate measures were made for each community in each assay environment. In all cases, the ‘winner of competition’ won in 3/3 independent replicate populations.

social milieu during selection	winner of competition or identity of single-lineage community	selection regime	relative growth rate	lag time (h)	maximum absorbance reached in 7 days	fraction of replicate populations that reach carrying capacity in 7 days
multi-strain A	473	control	0.6855 ± 0.3063	34.48 ± 36.96	0.2082 ± 0.0999	0/3
multi-strain B	2137	control	0.8800 ± 0.0661	76.53 ± 20.31	0.2252 ± 0.0541	0/3
single-strain	2137	control	3.4738 ± 0.2923	46.69 ± 37.25	0.3135 ± 0.1015	1/3
single-strain	2699	control	3.6558 ± 2.0326	39.68 ± 31.58	0.8212 ± 0.3917	2/3
single-strain	473	control	0.7739 ± 0.1943	51.25 ± 35.64	0.2486 ± 0.0958	0/3
multi-strain A	2699	high	1.4696 ± 0.1580	16.77 ± 4.74	0.4720 ± 0.0445	2/3
multi-strain B	2699	high	1.2323 ± 0.3011	27.77 ± 8.01	0.3762 ± 0.0793	1/3
single-strain	2137	high	3.5590 ± 0.2451	19.98 ± 15.87	0.9731 ± 0.2037	3/3
single-strain	2699	high	0.9928 ± 0.2161	45.49 ± 21.22	0.3005 ± 0.0645	0/3
single-strain	473	high	4.5417 ± 0.2714	33.54 ± 23.83	1.0838 ± 0.2278	3/3
multi-strain A	2699	rising	3.3387 ± 1.2176	38.01 ± 20.95	0.8105 ± 0.2835	3/3
multi-strain B	2137	rising	1.5061 ± 0.2338	62.70 ± 23.34	0.3595 ± 0.0725	0/3
single-strain	2137	rising	1.2920 ± 0.0293	71.01 ± 23.85	0.3150 ± 0.1039	1/3
single-strain	2699	rising	3.1689 ± 1.5588	43.44 ± 27.95	0.7910 ± 0.3738	2/3
single-strain	473	rising	3.5510 ± 0.7692	21.87 ± 18.88	0.8749 ± 0.3784	3/3

### (d) Calculations and statistics

All statistical analyses were carried out using R [16]. Analyses of fitness and productivity use ANOVAs of general linear models. In all cases, the predictors are named as follows: ‘assay’ refers to the actual level of CO_2_ at which response variable was measured, and is either high CO_2_ or air; populations were always acclimated for 7 days prior to an assay. ‘Selection regime’ refers to the control, rising and high CO_2_ selection regimes. ‘Social milieu’ is either single-strain community or multi-strain community. Assay, selection regime and social milieu are all fixed factors. When testing the effects of various characters on the competitive ability of a strain, the final frequencies of the strains are used as the response variable.

The Price equation partition from Collins & Gardner [1] is used to partition the changes in absorbance of populations under the CO_2_-enrichment regimes into contributions from physiology, evolutionary change within strains and competitive interactions between strains. Note that competition is called ‘ecology’ in the Collins & Gardner [1] paper. Each clone (genotypes that arise during the experiment within a particular lineage) is assigned an index *j*, and every strain is assigned an index *i. I* is the set of strain indices and *J*_*i*_ is the set of clone indices in a strain *i*. The change in a community-average character (total absorbance), Δ*z*_total_ that occurs over the time of the selection experiment in the CO_2_-enrichment regimes is calculated as:
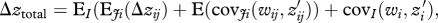

where E and cov are the statistical expectation (arithmetic average) and covariance, respectively, taken over the sets indicated by the subscripts; *z*_*ij*_ and 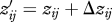 are the value of the character of interest (absorbance) exhibited by clone *j* in strain *i* in air and high CO_2_, respectively. *w*_*i*_ is the fitness of strain *i*. 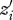 is the average value over all clones in a strain before enrichment, of the character value at high CO_2_. Details are given in appendix A and electronic supplementary material, table S1.

## 3. Results

### (a) Multi-strain and single-strain communities respond differently to selection

**Figure 1.**
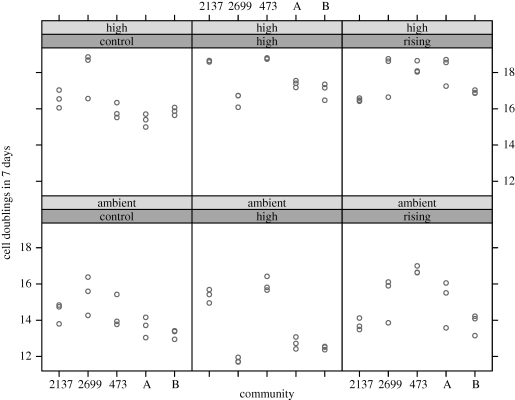
Number of cell doublings per 7 days in the evolved communities when grown in either high CO_2_ or in air. This corresponds to the time of a single transfer in the selection experiment, and was measured on a 96-well plate in the same conditions used for the selection experiment. Cell numbers were calculated from absorbance at 650 nm. The assay environment is denoted by either high (high CO_2_) or ambient. The selection regime is denoted by either control, high (high CO_2_) or rising (rising CO_2_). In all cases, communities were first acclimated for 7 days in the assay environment used for the data shown here. Each point represents the average of eight measurements of a single independent replicate community. Standard errors for each point range from 0.04 to 0.21.

Different strains have different direct evolutionary responses to selection, as expected from their initial differences in carbon uptake and fixation (strain × selection interaction on direct response to selection *F*_2,12_ = 19.41, *p* = 0.0002 for single-strain communities only, *F*_2,22_ = 22.95, *p* < 0.0001 for entire experiment; identity of winning strain used for multi-strain communities). In addition, the direct response to selection at rising and high CO_2_ differs between social regimes (effect of social milieu as single- or multi-strain on the direct response to selection; *F*_1,24_ = 18.28, *p* = 0.0003). The direct response to selection can be seen in figure 1, and is the fitness of the rising- and high-CO_2_ evolved communities grown in high CO_2_ relative to the control communities made up of the same strains, also assayed in high CO_2_. The end fitness also differs between single-strain communities and multi-strain communities (effect of social milieu; *F*_1,37_ = 9.02, *p* = 0.0048), so that within each individual selection regime, single- and multi-strain communities respond to selection differently.

### (b) Strains that are poor adapters when alone are good competitors when grown with other strains

Table 1 shows the outcome of competitive exclusion in multi-strain communities. In all cases save one, multi-strain communities are monomorphic by the end of the selection experiment. In the one case where the community is not monomorphic, the frequency of the rare type was less than 0.05. The same strain always won all three replicate competitions for a given selection regime and starting community, though the identity of the winning strain differed between regimes and starting communities. The difference in competitive outcomes between selection regimes suggests that particular competition winners are good competitors only in specific selective regimes, rather than good competitors in all environments. For example, 2699 wins all competitions in the high-CO_2_ selection regime, whereas 2137 wins all competitions when it is initially present in the rising CO_2_ and the control selection regimes. This pattern may indicate tradeoffs in competitive ability between environments.

So which measurements, if any, made in single-strain communities predict who will win a competition that goes on for hundreds of generations in a novel or changing environment? To test which traits measured in single-strain experiments may predict competitive ability in multi-strain experiments, I used fitness data combined with the outcomes of competitions in the two CO_2_-enrichment regimes described above. Though predictions about competition in high CO_2_ have been based either on ancestral fitness in elevated CO_2_ or on the ability of the ancestor to respond either physiologically or evolutionarily to CO_2_ enrichment [2,11], these factors are poor predictors of competitive outcome in this experiment. For instance, the ability of the ancestor to respond physiologically to changes in CO_2_ (measured as the fitness of the ancestor at high CO_2_ relative to its fitness in air) does not predict whether a strain wins a competition under CO_2_ enrichment (*t*_1_ = −0.85, *p* = 0.55). Similarly, the fitness of the ancestor at elevated CO_2_ does not predict whether a strain wins a competition under CO_2_ enrichment (*t*_1_ = 0.73, *p* = 0.60). Likewise, life-history traits that may be intuitively associated with competitive ability are poor predictors of competitive success. For instance, ancestral growth rate at high CO_2_ does not predict competitive success (*t*_1_ = 0.27, *p* = 0.83), although this is not surprising, since the data show that competitive exclusion is too slow to be attributed to initial differences in growth rates or fitness alone. Moreover, the final fitness of the single-strain community evolved in a given selection regime does not predict the outcome of competition in that selection regime (*t*_12_ = 1.16, *p* = 0.27). This makes sense: there is no reason that the end fitness of strains grown alone should necessarily predict long-term competitive ability.

The results reported above indicate that single-strain communities with high fitness at elevated CO_2_, either at the beginning or at the end of evolution, are not the ones that systematically win competitions in rising or high CO_2_ environments. They also indicate that life-history traits associated with being a good competitor, when present in the ancestor at the beginning of the experiment, do not predict the outcome of competition over hundreds of generations in multi-strain communities. Instead, the best predictor of competitive ability during an environmental change that can be measured in experiments done with single-strain communities is the direct response to selection of single-strain communities, which captures a strain's potential to adapt to abiotic change in the absence of competitors (*t*_13_ =− 5.89, *p* = 0.0001). Because strains with the weakest response to selection in specific environments when they are grown in the absence of competitors are the best competitors in those same environments when they are part of a multi-strain community, there is an apparent tradeoff between the potential for adaptation to abiotic change alone and competitive ability in this experiment.

The data here do not support the hypothesis that the winner of a competition can be predicted by examining the phenotype of the non-evolved ancestors in cases where within-lineage evolutionary change and between-lineage competition happen simultaneously. Of course, it is always possible that predictors such as ancestral physiological responses had weak abilities to predict the outcome of competition that were not detected because of low statistical power. That being said, the differences in *p*-values between the non-significant predictors and the significant predictor are large, and consistent with the qualitative outcomes of this experiment. For example, the 2699 lineage consistently wins competitions during evolution in the high-CO_2_ selection regime, yet the ancestral 2699 strain has the weakest physiological response to CO_2_ enrichment. Note that although the 2699 lineage has the ancestor with the highest fitness at elevated CO_2_, it also has the ancestor with the highest fitness in air. If ancestral fitness determined the chances of winning a competition, 2699 should have won all of the competitions, including those in the control selection regime. Instead, it wins only half of all competitions, which is barely better than might be expected by chance. However, 2699 shows almost no direct response to selection in the high-CO_2_ regime. A similar argument can be made for ancestral responses to changes in CO_2_ levels.

Further support for a tradeoff between competition and adaptation is seen in the conventional life-history components summarized in table 1. The lineages that evolve in multi-strain communities have lower maximum growth rates and lower maximum population sizes than the corresponding lineages that evolve in single-strain communities in the same selection regimes (effect of selection regime × social milieu: *F*_2,37_ = 2.91, *p* = 0.067, effect of assay environment × social milieu: *F*_1,39_ = 3.72, *p* = 0.061; control, rising and high selection regimes included in analysis. Effect of social milieu on maximum absorbance reached over 7 days: *F*_1,37_ = 6.501, *p* = 0.0006). Social milieu does not affect lag time (effect of social milieu on lag time *F*_1,37_ = 2.4918, *p* = 0.12). Since a lower growth rate coupled with a lower maximum population size must decrease the fitness of a lineage grown alone in batch culture, it is probable that competition limits how well adapted the eventual winners are in the final environment, once they have excluded competitors.

### (c) Competition decreases productivity

**Figure 2.**
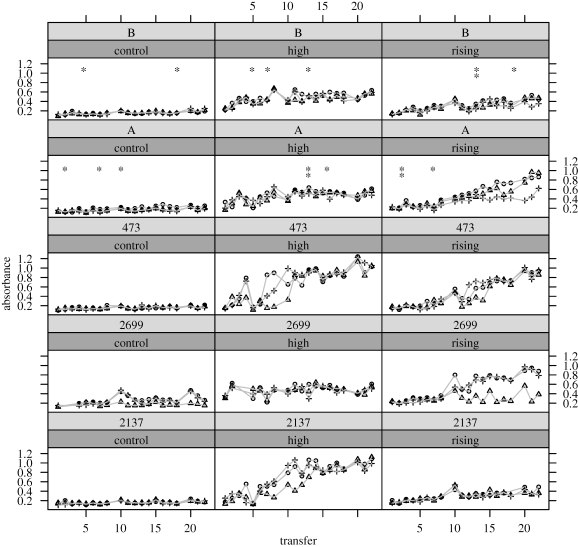
Absorbance of communities at the end of each transfer during the selection experiment. The approximate times where competitive exclusion occurred in multi-strain communities are indicated; DNA was extracted every other transfer and asterisks mark the last time that the ‘losing’ strain was detected. Each plot is a combination of community composition and selection regime. The top band above each panel shows the strains present in the community (community A is composed of the two mutants, 2699 and 473, while community B is composed of all three strains). The lower band above each panel shows the selection regime. Selection regimes are control, high (high CO_2_) and rising (rising CO_2_). The absorbance is the average of all 96 wells of a 96-well plate. Each point represents a single community (three replicate communities per selection regime). Absorbance is linearly related to cell number over the values used.

Treating adaptation within strains in the absence of competitors separately from competition between strains gives us insight into changes in community characters (besides fitness) that may be interesting for ecological, economic or other practical reasons. Here, I examine how adaptation and competition contribute to changes in productivity of microalgal communities under long-term CO_2_ enrichment. Figure 2 shows changes in absorbance, a conventional proxy for changes in biomass [17,18] in each community during the selection experiment. The framework in Collins & Gardner [1] uses the Price Equation to partition the relative contributions of physiology, competition and adaptation to changes in a population character. The results of this partition are given in table 2. Details of calculations are given in §2 and sections in the electronic supplementary material.

**Table 2.** Summary of Price equation partition. Summary of the contributions of physiology, evolution and competition to changes in absorbance of communities in the high- and rising-CO_2_ selection regimes. The units are simply average community absorbance at 650, measured as the mean of all 96 wells on a 96-well plate. Absorbance is related to cell number over the values used.

selection	community	winner	total difference in phenotype	physiological contribution	evolutionary contribution	ecological contribution
high	2137	n.a.	0.7389	0.18	0.5589	0
high	2699	n.a.	0.01521	0.6125	−0.5973	0
high	D473E	n.a.	0.8143	0.0967	0.7176	0
high	A	2699	0.155	0.3546	0.06015	−0.2598
high	B	2699	0.2163	0.2964	0.2264	−0.3066
rising	2137	n.a.	0.2083	0.18	0.02833	0
rising	2699	n.a.	0.5125	0.6125	−0.1	0
rising	D473E	n.a.	0.7417	0.0967	0.645	0
rising	A	2699	0.4978	0.3546	0.2725	−0.1293
rising	B	2137	0.2609	0.2964	0.1911	−0.2266

By the end of the experiment, all communities respond to long-term CO_2_ enrichment by producing more biomass than communities of equivalent composition in the control regime (high selection regime *t*_14_ = 5.74, *p* < 10^−4^, rising selection regime *t*_14_ = 3.82, *p* = 0.002, paired *t*-test, single tailed; values were an average of 4.1 times more under selection in rising CO_2_ and 4.6 times more under selection in high CO_2_ by the end of the experiment). However, multi-strain communities that simultaneously undergo adaptation and competition respond to a given selection regime differently than do single-strain communities (effect of social milieu on end absorbance, *F*_1,37_ = 4.43, *p* = 0.04; includes control, rising and high selection regimes). This implies that competition affects the production of biomass in a community.

The most striking result from the Price Equation partition is that competition in multi-strain communities always makes a large and a negative contribution to community absorbance during long-term growth under CO_2_ enrichment. This result suggests that multi-strain communities generally have lower biomass owing to the negative effect of competitive interactions (or their outcomes) on biomass. The change attributed to competition in this partition captures the tendency of more productive strains to increase in relative abundance when competing in communities. Since the term is negative, predictions about changes in community biomass that are made on the basis of individual components could overestimate biomass. This also indicates that biomass production is unlikely to be a good predictor of the success of particular strains within a changing community.

In cases where the partitioned term for evolution is positive and the partitioned term for competition is negative, natural selection is acting in different directions within and between strains. This sign difference is consistent with a tradeoff between adaptation to the abiotic environment and competitive ability. An important practical implication of the sign difference between evolution and competition terms is that they can cancel each other out when they are of similar magnitude, so that it may look as if changes in community productivity (or other phenotype) are due only to a sustained physiological response. Failing to note the contributions of evolution within strains and ecological interactions between them may, therefore, seriously undermine our ability to understand the underlying causes of changes in primary production under CO_2_ enrichment.

## 4. Discussion

Microbial experimental evolution can provide general insights into long-term processes. The main insight of this study is that strains with the least potential to adapt to a particular environment in the absence of competitors consistently make the best competitors in the face of that same environmental change when adaptation and competition can occur simultaneously. Moreover, given that all three strains used here *can* evolve to be good competitors in at least one of the studied environments, it is striking that single-strain experiments in those environments can predict which one actually *does* evolve to be a good competitor.

I suggest that this predictive power may be attributed to a tradeoff between competitive ability and the ability to adapt to abiotic change. While my proposed explanation of a tradeoff existing between adaptation within strains and competition between them is speculative, it is the most parsimonious general explanation of the outcome of this experiment. One practical example of when this tradeoff may be evident is that multi-strain communities are less productive than single-strain ones here, where the decrease in productivity is due to the negative effect of competition between strains.

Specific cases where a tradeoff is likely can be seen in this experiment. For example, single-strain cultures of 2699 do not adapt much in response to CO_2_ enrichment. However, when selected in a multi-strain community under CO_2_ enrichment, the evolved 2699 strains have higher growth rates than they do if they are selected alone (table 1), suggesting that they have instead evolved to be good competitors. This life-history strategy is not a general feature of 2699; when it evolves in a single-strain community, it does not have particularly high growth rates or short lag times compared with other single-strain communities in the same selection regimes. That strains selected in competition evolve to be good competitors is not surprising; what is surprising is the observation that the outcome of competition is repeatable within selection regimes, and that while all three strains can evolve life-history strategies that allow them to win competitions in some subset of environments, the strain that actually does evolve to be the best competitor in any particular environment is the one that evolves least in response to that abiotic selective pressure alone.

Fundamental relationships between processes such as adaptation and competition are general when they are described in terms of fitness rather than in terms of particular biology or life-history strategies. Single-strain communities evolving in response to environmental change have a single task (increase fit to abiotic environment), while strains in multi-strain communities have two tasks (increase fit to abiotic environment and outcompete other strains), which may slow the rate of adaptive evolution. An analogous scenario compares adaptation in more or less complex organisms, where increasing organismal complexity slows the rate of adaptive evolution [19]. This situation can be visualized as an *n*-dimensional adaptive landscape, where each axis represents an independent trait under selection, and fitness is mapped onto particular combinations of trait values. As organismal complexity increases, so does the number of axes (traits) that make up the adaptive landscape, which slows progress towards fitness optima. I propose that if competition and adaptation select on sets of traits that are not completely overlapping, then adding the task of competing while already adapting to abiotic change effectively increases the number of axes making up the adaptive landscape for an evolving population. While the particular situation will define the quantitative relationship between adaptive and competitive traits under selection, the expectation that the relationship will be positive is a reasonable place to start.

## 5. Conclusions

Here, I have demonstrated a way to use empirical information from evolution in single-strain communities to predict the outcome of competition between those strains in changed environments. I have also proposed a theoretical explanation for why this prediction works. Empirically, I have shown that the best predictor of a strain's competitive ability is its inability to adapt to abiotic change alone. Theoretically, I have suggested that a cost of complexity creates an intrinsic tradeoff between adaptation to abiotic change and competitive ability. This theoretical framework could allow the relationship between competitive interactions and adaptation to abiotic environmental change to be modelled without *a priori* information about the specific form of ecological interactions. While the idea of an intrinsic tradeoff between adaptive evolution within strains and competitive ability between them is speculative, it is the most parsimonious general explanation of the outcome of this experiment. Future work must independently test this idea in other model systems and under various conditions to investigate its generality.

I have used experimental evolution to investigate the interplay between adaptive evolution and ecological competition when competitive interactions and adaptation to environmental change occur simultaneously. I have used particular conditions that test the hypothesis that differences in physiological response to CO_2_ enrichment predict competitive outcomes for photosynthetic microbes. I have shown that the outcome of competition can be predicted from the evolved phenotype of single-strain communities, where strains that are least able to respond to CO_2_ enrichment when grown in isolation are the ones that are best able to exclude competitors as they evolve in a multi-strain community. This finding implies that we should revisit predictions of community composition and productivity that are based on the assumption that the types best able to respond to elevated CO_2_ will increase in frequency when in communities. While my findings do not apply to cases where the outcome of competition is determined by gross differences in biology between functional types of phytoplankton, they are relevant to understanding how the species or lineage composition within functional groups, and therefore the characters of particular functional groups, are likely to change. In the particular case of marine phytoplankton, it is especially important to make good use of common laboratory model systems to draw general conclusions about long-term responses to environmental change, given the difficulty of doing well-replicated, generalizable microbial evolution experiments using marine phytoplankton.

## Appendix A. Details of Price Equation Partition

The first term, E_*I*_(E_*Ji*_(Δz_*ij*_)), is the contribution of physiology. The second term,  is the contribution of evolution, which denotes the tendency of clones with a higher or lower character value to increase or decrease in relative abundance within strains. The last term, , is the contribution of competition, which shows the tendency of strains with a higher or lower character value to increase in abundance, that is, to be good competitors. The total difference in phenotype (Δ*z*_total_), is simply the difference in average absorbance per well of a community between the end and the beginning of the experiment. Absorbances were calculated using the absorbances measured at the first and last three transfers, and are the averages of three independent communities. The physiology term is (final absorbance) – (initial absorbance) of the control communities when they are grown at elevated CO_2_. Strictly speaking, this term should be calculated from the physiological responses of the ancestral populations. However, the Price equation maps entities (in this case clones) onto each other between populations. One important approximation that I make in applying this partition to my data is that since the end populations are large and originate from a common ancestor, they have sampled the same mutations, such that the same subset of mutants are present in the control populations and those selected under CO_2_ enrichment that originate from the same ancestor, even though the particular clone that dominates one population may only be present at mutation–selection balance in the other population. Because of this approximation, the control populations, which have experienced mutation, but no environmental change, rather than the ancestors, who have not had time to sample many mutations, are used to calculate the physiology term. The competition term is (by definition) zero in the single-strain communities. The expectation of the evolution term is the total change in phenotype minus the contribution of physiology. The evolution term has a covariance that was not directly measured in this experiment, and is calculated from the partition in the single-strain populations, since all other terms are known. The covariance term for each lineage under each selection regime for CO_2_ enrichment is used in the partition for the multi-strain communities. In this case, the ecology term is calculated by difference. Briefly, the total change in phenotype is as for the single-strain communities. The physiology term is calculated as above for each lineage present in the starting population and a weighted average is taken. The evolution term is a weighted average of the product of the fitnesses of the single-strain populations and the covariance term calculated for each lineage from the single-strain communities. A breakdown of specific values used for calculations is given in the electronic supplementary material, table S1*a*,*b*.
